# PRRSV evades innate immune cGAS-STING antiviral function via its Nsp5 to deter STING translocation and activation

**DOI:** 10.1080/21505594.2025.2548625

**Published:** 2025-08-21

**Authors:** Yulin Xu, Chenglin Chi, Qihang Xin, Jiang Yu, Yuyu Zhang, Pingping Zhang, Wangli Zheng, Sen Jiang, Wanglong Zheng, Nanhua Chen, Jiaqiang Wu, Jianzhong Zhu

**Affiliations:** a Key Laboratory of Livestock and Poultry Multi-omics of Ministry of Agriculture and Rural Affairs (MARA), Institute of Animal Science and Veterinary Medicine, Shandong Academy of Agricultural Sciences, Jinan, China; b College of Veterinary Medicine, Yangzhou University, Yangzhou, China; c Joint International Research Laboratory of Agriculture and Agri-Product Safety, Yangzhou University, Yangzhou, China; d Comparative Medicine Research Institute, Yangzhou University, Yangzhou, China; e Jiangsu Co-Innovation Center for Prevention and Control of Important Animal Infectious Diseases and Zoonoses, Yangzhou University, Yangzhou, China

**Keywords:** PRRSV, Nsp5, STING, immune evasion

## Abstract

Porcine Reproductive and Respiratory Syndrome Virus (PRRSV) is an important pathogen that seriously endangers pig breeding, causing significant economic losses to the global swine industry. Our previous study found that the DNA sensing innate cGAS-STING signaling pathway plays an important role in inducing interferon (IFN) upon PRRSV infection and inhibition of PRRSV replication. However, the mechanism underlying immune evasion by PRRSV remains unclear. In the current study, we found that PRRSV non-structural protein 5 (Nsp5) strongly inhibits the cGAS-STING-IFN antiviral response. Furthermore, we found that Nsp5 interacts with STING, blocking STING transport from the ER to the Golgi apparatus and interfering with STING recruitment of TBK1/IKKε/IRF3. Finally, we demonstrated that the Nsp5 36–47 and 58–67 amino acid regions are critical for inhibiting STING activity and PRRSV replication. This study describes a novel mechanism by which PRRSV suppresses the host innate antiviral response and has implications for our understanding of PRRSV pathogenesis.

## Introduction

Porcine reproductive and respiratory syndrome (PRRS) is an economically important viral disease worldwide characterized by reproductive failure in sows and respiratory diseases in growing pigs [1]. PRRS was first reported in the United States in 1987 and in China in 1996 [2]. The pathogenic agent of PRRS is the porcine reproductive and respiratory syndrome virus (PRRSV), a positive-sense single-stranded RNA virus. It belongs to the family *Arteriviridae*, and can be divided into two species: PRRSV-1 (prototype European Lelystad virus LV strain) and PRRSV-2 (prototype North American VR2332 strain) [3]. PRRSV is an enveloped virus with a genome approximately 15.4 kb in length and contains at least 10 open reading frames (ORFs) expressing non-structural polyproteins encoded by ORF1a and ORF1b, and eight structural proteins encoded by ORF2–7 (GP2, E, GP3, GP4, GP5, ORF5a, M, and N) [4]. Viral polyproteins are generated during infection by differential ribosome frameshift (RFS) events at two genomic sites, including pp1a, pp1a-nsp2N, pp1a-nsp2TF and pp1ab. These polyproteins are cleaved into at least 16 non-structural proteins (Nsps) composed of Nsp1α, Nsp1β, Nsp2N, Nsp2TF, Nsp2-Nsp6, Nsp7α, Nsp7β, Nsp8, Nsp9 to Nsp12, by viral proteases nsp1α, nsp1β, nsp2 and nsp4, collectively [4]. PRRSV non-structural proteins play an important role in supporting viral replication and modulating the host response [4–6].

Innate immunity serves as the first-line host defense against infections and utilizes multiple pattern recognition receptors (PRRs) to monitor invading pathogens by sensing pathogen-associated molecular patterns (PAMPs) and damage-associated molecular patterns (DAMPs) [7]. Innate immune PRRs include Toll-like receptors (TLRs), RIG-I-like receptors (RLRs), NOD-like receptors (NLRs), C-type lectin-like receptors (CLRs) and cytosolic DNA receptors (CDRs) [8–10]. Stimulator of interferon genes (STING) plays a pivotal role in innate immune responses as the central adaptor for DNA sensors, with cGAS being the most defined [11,12]. Upon DNA sensing, cGAS catalyzes the synthesis of the second messenger 2’3’-cGAMP, which activates STING in the endoplasmic reticulum (ER) [12]. Activated STING transfers from the ER to the trans-Golgi apparatus network, where it recruits TBK1, IKKε, and the subsequent transcription factor IRF3 to form a signalosome, by which both TBK1 and IKKε phosphorylate IRF3 [12–14]. Phosphorylated IRF3 then translocates into the nucleus to activate the expression of antiviral type I interferons (IFNs), orchestrating innate immune responses [12].

STING has been shown to play a key role in sensing DNA viral infections, such as herpes simplex virus 1 (HSV-1) [15], human papillomavirus [16], pseudorabies virus (PRV) [17], and African swine fever virus [18]. In addition, STING plays an important role in host resistance against RNA virus infections, such as Hanta virus [19], Zika virus [20] and PRRSV [21]. At the same time, viruses have evolved corresponding antagonizing machinery to evade the host antiviral immune defense. PRV utilizes its tegument protein UL13 to recruit RNF5 and inhibit STING-mediated antiviral immunity [17]. Zika virus evades the STING antiviral response by cleaving cGAS through the NS1-caspase-1 axis [20]. 3 CL of SARS-CoV-2 inhibits K63-ubiquitin modification of STING to disrupt the assembly of the STING functional complex and downstream antiviral signaling [22]. PRRSV, an RNA virus, has developed multiple mechanisms to antagonize the RNA-sensing innate immune antiviral response [5,6]. However, it remains unclear whether and how PRRSV evades the DNA-sensing cGAS-STING antiviral pathway.

In the present study, we screened PRRSV proteins to interact with porcine STING and found that viral non-structural protein 5 (Nsp5) was able to interact with STING and suppress STING activation by deterring its translocation from the ER to the Golgi, and recruitment of downstream TBK1/IKKε/IRF3. These findings have implications for PRRSV pathogenesis and reveal potential targets for the development of novel therapeutic strategies against PRRSV.

## Materials and methods

### Cells and viruses

Marc-145 (Ubigene# YC-A070), BHK-21 (ATCC# CCL-10) and HEK-293T (ATCC# CRL-3216) cells were cultured in Dulbecco’s modified Eagle’s medium (DMEM; Gibco, Grand Island, NY, USA) containing 10% fetal bovine serum (FBS, Gibco), 100 U/mL penicillin and 100 μg/mL streptomycin. Porcine alveolar macrophages (3D4/21, ATCC# CRL-2843) and STING^−/−^ 3D4/21 cells [23] were cultured in RPMI 1640 medium containing 10% FBS and penicillin/streptomycin. All the cells were cultured at 37°C in a humidified atmosphere containing 5% CO_2_. The PRRSV strain used in this study was the highly pathogenic PRRSV XJ17-5 (PRRSV-2, GenBank ID: MK759853.1) isolated and preserved in our laboratory [24]. The virus was propagated and titrated in Marc-145 cells grown in DMEM supplemented with 2% FBS.

### Antibodies and reagents

Mouse FLAG mAb (HT201), GFP mAb (HT801) and actin mAb (HC201) were acquired from TransGen Biotech (Beijing, China). Rabbit GFP mAb (AE078), mouse HA mAb (AE008), mouse GAPDH mAb (AC002) and rabbit histone 3 H3 pAb (A2352) were purchased from ABclonal (Wuhan, Hubei, China). Rabbit STING (19851-1-AP), anti-GORASP2 (10598–1-AP) and Myc (16286-1-AP) pAbs were purchased from ProteinTech (Wuhan, Hubei, China). Rabbit mCherry (ab183628) and Calreticulin (ab2907) pAbs were acquired from Abcam (Cambridge, Cambridgeshire, UK). Rabbit TBK1 mAb (3504S), phosphorylated-TBK1 (p-TBK1, 5483S) mAb (5483S), IRF3 mAb (11904S), and HA mAb (3724) were acquired from Cell Signaling Technology (Boston, MA, USA). The phosphorylated-IRF3 (p-IRF3, Ser385) rabbit pAb (MA5–14947), Lipofectamine 2000 and Lipofectamine 3000 were purchased from Thermo Fisher Scientific (Sunnyvale, CA, USA). The ClonExpress MultiS One Step Cloning Kit (C113) was purchased from Vazyme Biotech (Nanjing, Jiangsu, China). Protein A/G PLUS-Agarose was purchased from Santa Cruz Biotechnology (sc-2003, CA, USA). 2’3’-cGAMP was purchased from InvivoGen (Hong Kong, China).

### Construction of recombinant expressing plasmids

Total RNA was extracted from HP-PRRSV XJ17–5 using TRIpure reagent, and 22 open reading frames (ORFs) of PRRSV were amplified by RT-PCR using the primers listed in Table S1. PCR products were inserted into the *Nhe*I and *Age*I sites, *Nhe*I and *EcoR*V sites or *Xho*I and *EcoRV* sites of the pEGFP-N1 vector. For 10 truncated Nsp5 mutants (amino acids 1–101, 1–67, 1–57, 1–47, 1–35, 102–170, 68–170, 58–170, 48–170 and 36–170), the gene fragments were each PCR amplified from pEGFP-N1-Nsp5 using the primers listed in Table S2 and then cloned into the *Nhe*I and *Age*I sites of the pEGFP-N1 vector. For Nsp5 deletion mutants, the two flanking sequences of deletion sites were separately PCR amplified, and then two PCR products were fused by overlapping PCR. Fusion PCR products were cloned into the *Nhe*I and *Age*I sites of the pEGFP-N1 vector. All the PCR primers used are listed in Table S2. For porcine STING 1–153, the sequence of amino acids 1-153 was amplified by PCR from the pmCherry-C1-pSTING plasmid using the primers listed in Table S3, and cloned into the *Bgl*II and *EcoR*I sites of the pmCherry-C1 vector. All plasmid constructs were confirmed using Sanger DNA sequencing.

The pcDNA-DEST-pcGAS-2 HA, pcDNA-DEST-pSTING-2 HA, pEGFP-C1-pSTING, pmCherry-C1-pSTING, pcDNA-DEST-pSTING-2 HA (amino acids 1-190, 191-378, 1-339 and 151-339), pcDNA-Myc-TBK1, pcDNA-Myc-IKKε and p3×FLAG-CMV-7.1-pIRF3 were cloned and stored in our laboratory.

### Western blotting and co-immunoprecipitation

Western blotting was performed as we described previously [21]. Briefly, whole cell protein was extracted and separated by SDS-PAGE, and the protein in the gel was transferred to a PVDF membrane. The membrane was blocked, incubated with the indicated primary antibodies at 4°C overnight, and then incubated with HRP-labeled anti-rabbit or anti-mouse antibodies (1:1000) (Sangon Biotech, Shanghai, China). The protein signal was visualized with a Western blot imaging system using an ECL chemiluminescent detection system (Tanon, Shanghai, China).

For co-immunoprecipitation (Co-IP), the cell lysate from transfected cells in 6-well plate (6–8 × 10^5^ cells/well) was incubated with 1 μg of specific antibodies at 4°C overnight with shaking and further incubated with Protein A/G PLUS-Agarose for 2–3 h. The beads were washed thrice with RIPA buffer and eluted with 2×SDS sample buffer. The elution samples, together with the input lysate controls, were subjected to Western blotting.

### Confocal microscopy

3D4/21 cells in 24-well plates were transfected with the indicated plasmids and then fixed with 4% paraformaldehyde for 30 min. Fixed cells were permeabilized with 0.5% Triton X-100 for 20 min. Next, the nuclei were stained with 4′, 6-diamidino-2-phenylindole (DAPI) for 15 min.

To detect the traffic of STING, mCherry-STING, Nsp5-GFP, Nsp5-GFP (Δ36-47), Nsp5-GFP (Δ58-67), Nsp5-GFP (Δ36–47Δ58–67) or an empty vector was transfected into Marc-145 cells and then treated with or without 2’3’-cGAMP. After 24 h, the cells were incubated with antibodies (rabbit anti-calreticulin or rabbit anti-GORASP2) for 1 h at 37°C. The cells were incubated with Alexa Fluor 647-conjugated goat anti-rabbit IgG (H+L) (A32733, Thermo Fisher, USA) for 1 h at 37°C in the dark, and the cell nuclei were stained with DAPI for 15 min. Images were visualized using laser-scanning confocal microscope (LSCM, Leica SP8, Solms, Germany).

### Promoter driven luciferase reporter gene assays

HEK-293T cells grown in 96-well plates (3 × 10^4^ cells/well) were co-transfected using Lipofectamine 2000 with a firefly luciferase (Fluc) reporter plasmid at 10 ng/well (ISRE-Fluc, ELAM (NF-κB)-Fluc or IFNβ-Fluc) and β-actin *Renilla* luciferase (Rluc) reporter (0.2 ng/well), together with the expression plasmids (20, 50 or 70 ng/well). The total amount of DNA was maintained constant by adding a vector control plasmid. At 24 h post transfection, the cells were lysed and samples were assayed as we described previously [25].

### RNA extraction and reverse transcription-quantitative PCR

Total RNA was extracted from HEK-293T cells in 24-well plates with TRIpure Reagent, and cDNA synthesis was performed using HiScript® 1st Strand cDNA Synthesis Kit (+gDNA wiper, Vazyme Biotech). The quantitative PCR was then performed with ChamQ Universal SYBR qPCR Master Mix using StepOne Plus equipment (Applied Biosystems) to measure the target gene expressions. The qPCR program is denaturing at 95°C for 30 s followed by 40 cycles of 95°C for 10 s and 60°C for 30 s. The relative ISG56, ISG60 and IFNβ mRNA levels were normalized to RPL32 mRNA levels and calculated using the 2^−∆∆Ct^ method. The sequences of qPCR primers used are shown in Supplemental Table S4.

### Isolation of cytosolic and nuclear proteins

Cells in 12-well plates were transfected with the Nsp5 plasmid for 24 h and then stimulated by transfection with 2’3’-cGAMP for 12 h. After treatment, cytosolic and nuclear proteins were isolated using a Nuclear and Cytoplasmic Protein Extraction Kit (Beyotime, Shanghai, China) following the manufacturer’s instructions and as we previously described [26].

### Construction of recombinant PRRSVs with Nsp5 mutations

To generate PRRSV infectious clones carrying Nsp5 deletions, the full-genome cDNA clones of pACYC177-CMV-rXJ17–5 and pACYC177-CMV-rXJ17–5-EGFP that we constructed previously [27,28] were used as skeletons. Fragment 1 (F1) of the two skeleton plasmids were replaced with F1 with Nsp5 gene deletions. Recombinant PRRSV infectious clones pACYC177-CMV-rXJ17–5/EGFP-Nsp5Δ36–47, pACYC177-CMV-rXJ17–5/EGFP-Nsp5Δ58–67 and pACYC177-CMV-rXJ17–5/EGFP-Nsp5Δ36–47Δ58–67 were prepared.

### Statistical analysis

All the statistical analyses were performed with GraphPad Prism 6 software. Data were presented as the mean ± standard deviation (SD) from three independent samples. Statistically significant differences between groups were determined using the Student’s *t*-test. *p* < 0.05 was considered statistically significant. In the figures, “*” and “**” denote *p* < 0.05 and *p* < 0.01, respectively.

## Results

### The PRRSV Nsp3, Nsp5 and E proteins interact with porcine STING

**Figure 1.**
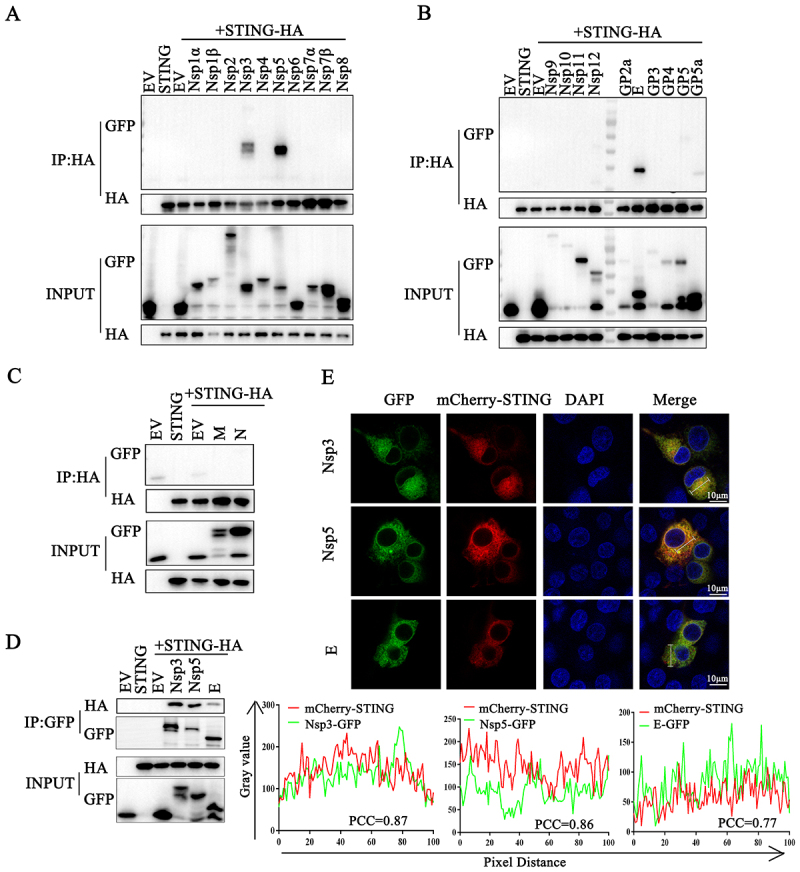
PRRSV proteins Nsp3, Nsp5 and E interact with STING. (A-C) HEK-293T cells were co-transfected pSTING-HA together with the indicated plasmids encoding GFP-tagged PRRSV viral proteins for 24 h. The cells were harvested for immunoprecipitation using rabbit anti-HA antibody, and IP samples and cell lysate inputs were subjected to Western blotting by using mouse anti-HA antibody and mouse anti-GFP antibody. (D) HEK-293T cells were co-transfected pSTING-HA together with the plasmids encoding GFP-tagged PRRSV viral proteins (Nsp3, Nsp5 and E) for 24 h, then harvested for immunoprecipitation using mouse anti-GFP antibody. The IP samples and cell lysate inputs were subjected to Western blotting by using rabbit anti-HA antibody and rabbit anti-GFP antibody. (E) 3D4/21 cells were co-transfected with mCherry-STING together with the indicated plasmid encoding GFP-tagged PRRSV viral proteins (Nsp3, Nsp5 and E) for 24 h, and examined for co-localization by confocal microscopy. The fluorescence intensity profile of mCherry (red) and GFP (green) was measured along the line, and the level of co-localization was quantified by calculating Pearson’s correlation coefficient (PCC) using ImageJ software. Scale bars of 10 µm are shown in the images.

As an RNA virus, the PRRSV genome is mainly recognized by innate immune RNA-sensing receptors; however, our previous study demonstrated that the innate immune DNA receptor cGAS-STING signaling pathway plays an important role in sensing and defense against PRRSV infection [21]. However, whether and how PRRSV evades the DNA-sensing cGAS-STING antiviral pathway remains unclear. Here, we focused on porcine STING and sought to examine the STING-interacting PRRSV proteins for inhibitory effect on cGAS-STING antiviral signaling. The Co-IP assay screening of all PRRSV proteins showed that the PRRSV proteins Nsp3, Nsp5 and E interact with porcine STING (Figure 1(A–C)). The reverse Co-IP (Figure 1(D)) and co-localization assays (Figure 1(E)) further confirmed these interactions. The results clearly showed that PRRSV Nsp3, Nsp5 and E proteins interact with porcine STING and potentially play a role in the immune evasion of STING antiviral signaling.

### The PRRSV protein Nsp5 inhibits the cGAS-STING-mediated type I IFN response

**Figure 2.**
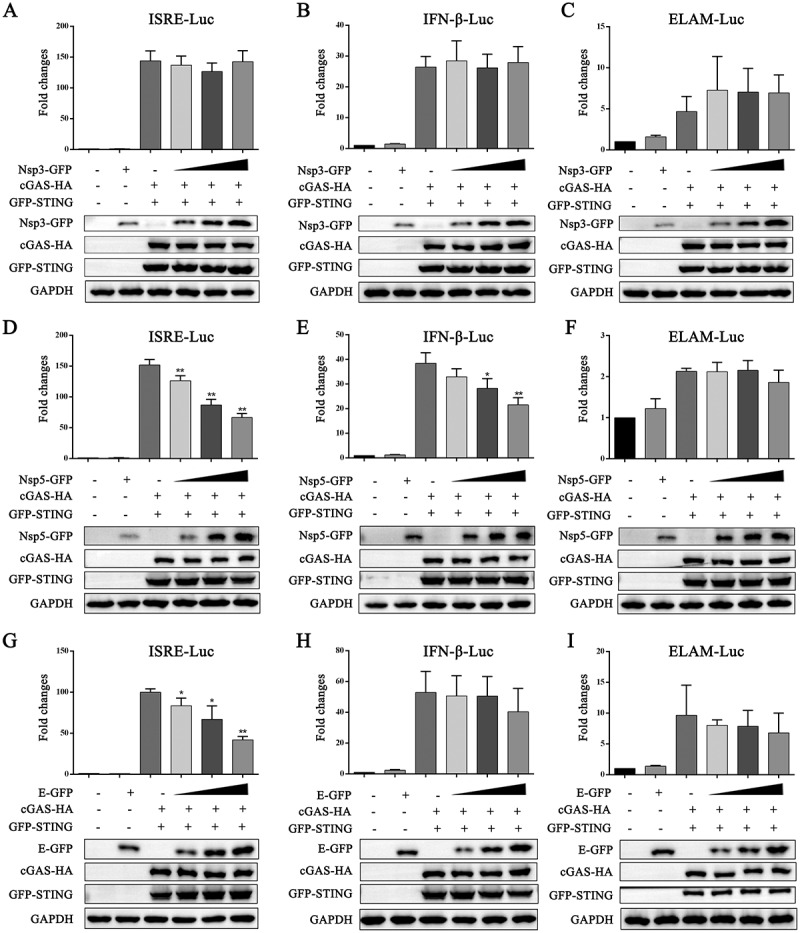
PRRSV Nsp5 inhibits the activity of the ISRE and IFNβ promoters mediated by the cGAS-STING pathway. HEK-293T cells grown in 96-well plate (3 × 10^4^ cells/well) were transfected with ISRE (A, D and G), IFNβ (B, E and H), or ELAM (C, F and I) (10 ng) luciferase reporter, *Renilla* (0.2 ng) reporter, and plasmids expressing cGAS (10 ng) and STING (10 ng), together with increase amounts (20, 50, and 70 ng) of plasmids expressing Nsp3, Nsp5 and E. The total DNA was normalized to 100 ng/well and the luciferase activities were measured at 24 h post transfection. The expressions of cGAS, STING, Nsp3, Nsp5, E and GAPDH were detected by Western blotting. *p < 0.05, **p < 0.01.

Furthermore, we evaluated innate immune modulation by PRRSV Nsp3, Nsp5 and E proteins using a luciferase reporter assay. We observed that Nsp3 did not inhibit the promoter activity of ISRE, IFNβ and ELAM mediated by the cGAS-STING pathway (Figure 2(A–C)). Nsp5 inhibited the activity of both ISRE and IFNβ promoters (Figure 2(D–F)), whereas the E protein only inhibited the activity of the ISRE promoter but did not inhibit the activity of IFNβ and ELAM promoters (Figure 2(G–I)). Similarly, only Nsp5 inhibited the cGAS-STING activated downstream IFNβ, ISG56 and ISG60 gene inductions (Figure S1). Therefore, we selected PRRSV Nsp5 for the subsequent experiments.

**Figure 3.**
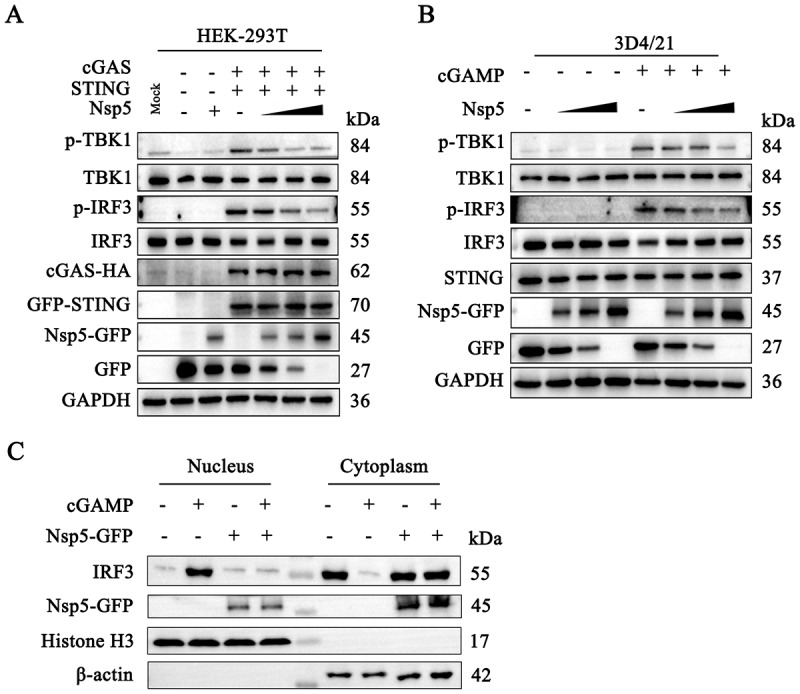
PRRSV Nsp5 inhibits the STING-mediated downstream signaling pathway. (A) HEK-293T cells in a 6-well plate were transfected with cGAS (0.5 μg), STING (0.5 μg) and Nsp5 (0.25, 0.5 or 1 μg) for 24 h. Empty vector was added to keep the total DNA amount constant. (B) 3D4/21 cells in a 12-well plate were transfected with Nsp5 (0.25, 0.5 or 1 μg) for 12 h, then cells were transfected with 2’3’-cGAMP (200 ng/mL) for 12 h. The cell lysates were subjected to Western blotting using the indicated antibodies. (C) 3D4/21 cells were transfected or not with Nsp5 (1 μg) for 24 h and then treated with or without 2’3’-cGAMP (1 μg/mL) for 12 h. Cells were then harvested for extraction of cytoplasmic and nuclear proteins. The extracted proteins were determined by Western blotting with antibodies against IRF3, GFP, histone H3, and β-actin, respectively.

To explore the impact of Nsp5 on the DNA-sensing cGAS-STING pathway, we analyzed the effects of Nsp5 on the activation of relevant signaling proteins in the cGAS-STING signaling pathway using Western blotting. In transfected HEK-293T cells, exogenous porcine cGAS-STING activated phosphorylation of TBK1 (p-TBK1) and IRF3 (p-IRF3), which were both inhibited by Nsp5 in a dose-dependent manner (Figure 3(A)). In porcine macrophage 3D4/21 cells, the levels of p-TBK1 and p-IRF3 induced by endogenous STING activation were inhibited by Nsp5 in a dose-dependent manner (Figure 3(B)). Furthermore, Nsp5 inhibited IRF3 nuclear translocation upon STING activation by 2’3’-cGAMP (Figure 3(C)). These results demonstrated that PRRSV Nsp5 invades STING-mediated type I IFN signaling.

### PRRSV Nsp5 inhibits STING signaling by retaining STING at the ER

**Figure 4.**
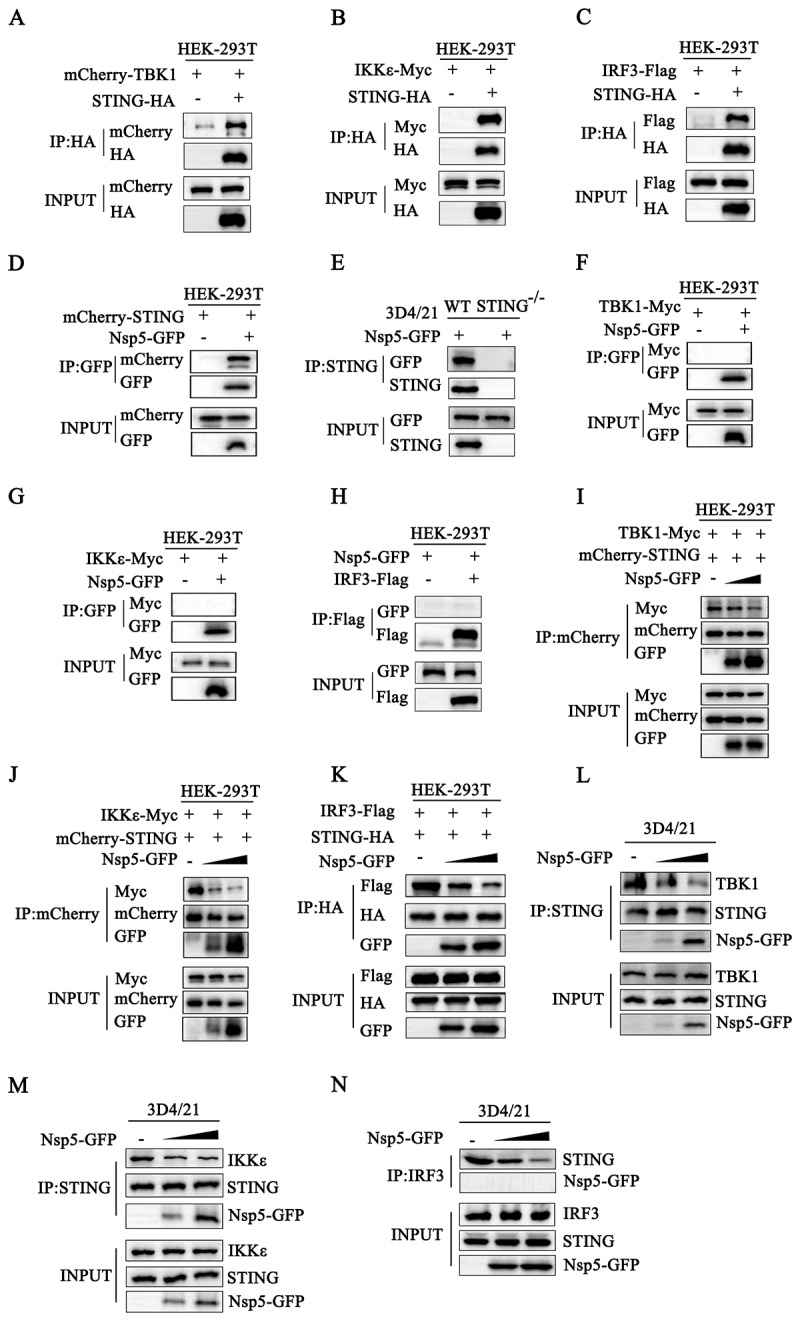
PRRSV Nsp5 inhibits the cGAS-STING pathway through its interaction with STING. (A-C) HEK-293T cells were co-transfected with mCherry-TBK1 and STING-HA, with IKKε-myc and STING-HA, and with IRF3-Flag and STING-HA for 24 h. (D) HEK-293T cells were co-transfected with mCherry-STING and Nsp5-GFP for 24 h. (E) STING-deficient 3D4/21 cells were transfected with Nsp5-GFP for 24 h. (F-H) HEK-293T cells were co-transfected with TBK1-myc and Nsp5-GFP (F), with IKKε-myc and Nsp5-GFP (G), and with IRF3-Flag and Nsp5-GFP (H) for 24 h. (I-K) HEK-293T cells were co-transfected with 0.5 μg mCherry-STING, 0.5 μg TBK1-myc, and increasing amounts of Nsp5-GFP (0.5 and 1 μg) for 24 h (I), with 0.5 μg mCherry-STING, 0.5 μg IKKε-myc and increasing amounts of Nsp5-GFP (0.5 and 1 μg) for 24 h (J), with 0.5 μg STING-HA, 0.5 μg IRF3-Flag and increasing amounts of Nsp5-GFP (0.5 and 1 μg) for 24 h (K). (L-N) 3D4/21 cells were transfected with Nsp5-GFP (1 and 2 μg), then stimulated with 2’3’-cGAMP for 12 h. The cells were harvested and subjected to Co-IP and Western blotting using the indicated antibodies.

Our recent study showed that STING recruits both TBK1 and IKKε during translocation from the ER to the Golgi apparatus, and that STING, TBK1, IKKε and IRF3 May form a signalosome by which both TBK1 and IKKε phosphorylate IRF3 and other substrates for the type I IFN response [13]. First, the interactions between porcine STING and three signaling proteins, porcine TBK1, IKKε and IRF3 were examined using a Co-IP assay. As expected, STING interacted with TBK1, IKKε and IRF3, respectively (Figure 4(A–C)). Although Nsp5 interacted with STING (Figure 4(D,E)), it did not interact with TBK1, IKKε or IRF3 (Figure 4(F–H)). However, Nsp5 was able to inhibit the interactions between STING and TBK1 (Figure 4(I)), STING and IKKε (Figure 4(J)), and STING and IRF3 (Figure 4(K)), in a dose-dependent manner. More importantly, Nsp5 was able to inhibit the interactions between endogenous STING and TBK1/IKK**ε**/IRF3 in a dose-dependent manner (Figure 4(L–N)).

**Figure 5.**
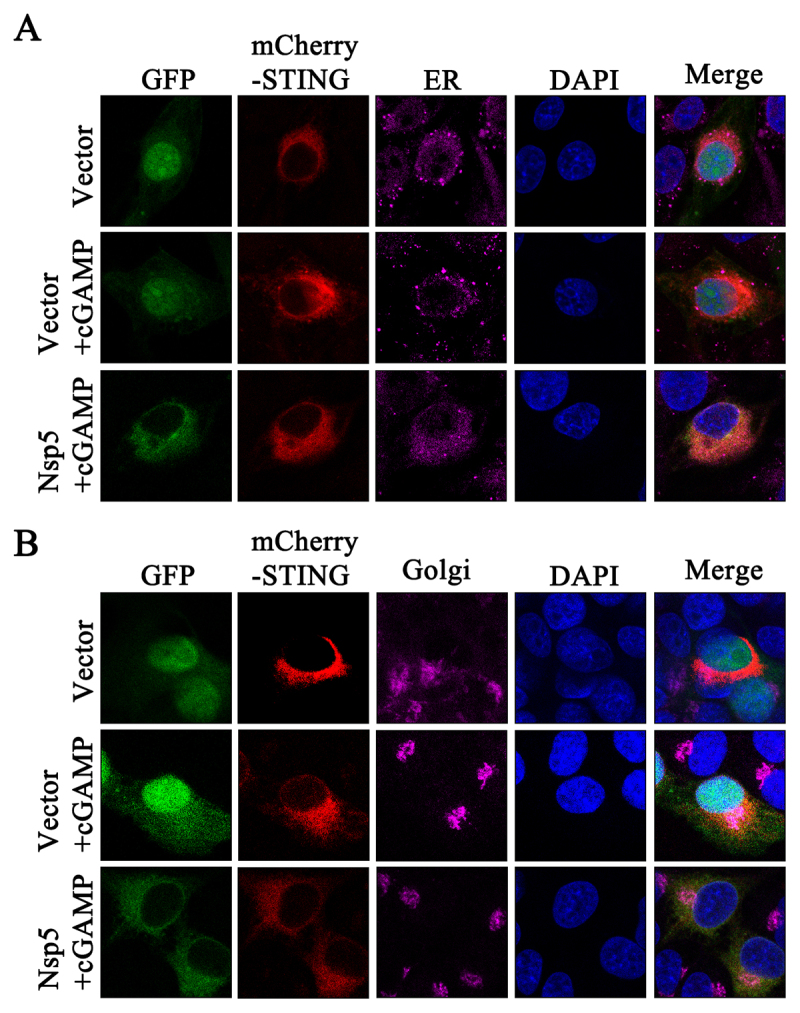
PRRSV Nsp5 blocks STING trafficking from the ER to the Golgi apparatus. (A and B) 3D4/21 cells were co-transfected with Nsp5 and STING expression plasmids for 24 h, and then treated with or without 2’3’-cGAMP for 12 h. Cells were fixed, permeabilized, and incubated with antibodies specific for Calreticulin (ER marker, A) and GORASP2 (Golgi marker, B), followed by counter-staining with DAPI. Samples were examined under a confocal microscope.

STING is an ER-resident protein, and its translocation from the ER to the Golgi apparatus is a hallmark of STING activation [29]. Considering that Nsp5 inhibits the cGAS-STING pathway through its interaction with STING and interference with the recruitment of TBK1, IKKε and IRF3, we hypothesized that Nsp5 May interfere with STING translocation from the ER to the Golgi apparatus. Therefore, ectopic Nsp5-GFP and mCherry-STING were expressed in porcine macrophages 3D4/21, followed by stimulation with 2’3’-cGAMP, and then the cells were processed for immunostaining to detect the STING location. As predicted, STING was located in the ER of unstimulated cells, and upon stimulation, it was transported from the ER to the Golgi apparatus (Figure 5(A)), forming large puncta in the Golgi apparatus (Figure 5(B)). In contrast, in Nsp5 expressed cells, STING remained in the ER and had no puncta formation upon 2’3’-cGAMP stimulation (Figure 5(A,B)). These data indicated that Nsp5 inhibits STING signaling by hindering STING trafficking.

### The 1–153 amino acids of porcine STING is a critical region for interaction with Nsp5

**Figure 6.**
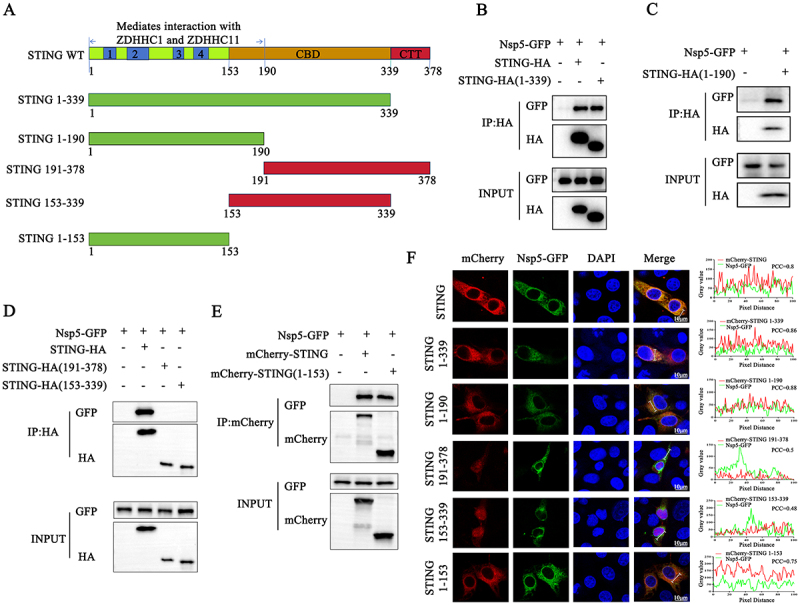
Porcine STING 1–153 amino acids is a critical region for interaction with Nsp5. (A) A schematic of porcine STING molecular structure and its truncated mutants. (B-E) HEK-293T cells were co-transfected with STING or its truncation mutants and Nsp5-GFP for 24 h, and the cells were harvested and subjected to Co-IP and subsequent Western blot analysis using the indicated antibodies. (F) mCherry-STING or its truncation mutants were co-transfected with Nsp5-GFP into 3D4/21 cells for 24 h. Cells were fixed, permeabilized, and examined for co-localization by confocal microscopy. The levels of co-localization between STING or its truncation mutants and Nsp5 were quantified by PCC using ImageJ software. Scale bars are10 µm.

To identify which domain of porcine STING is important for the interaction of STING with Nsp5, five truncated mutants of STING were prepared (Figure 6(A)). Subsequently, a Co-IP assay was used to identify the STING domains important for Nsp5 interaction. Domain mapping revealed that Nsp5 interacts with STING 1–339 and STING 1–190 (Figure 6(B,C)), but not with STING 191–378 and STING 153–339 (Figure 6(D)). Based on these results, we inferred that STING 1–153 is the key domain for STING interaction with Nsp5. As proposed, STING 1–153 is necessary and sufficient for the interaction of STING with Nsp5 (Figure 6(E)). Corresponding with these Co-IP results, mCherry-STING 1–153 co-localized very well with Nsp5-GFP in the cytoplasm of transfected cells (Figure 6(F)).

### The 36–47 and 58–67 amino acids of Nsp5 are the key regions for interaction with STING

**Figure 7.**
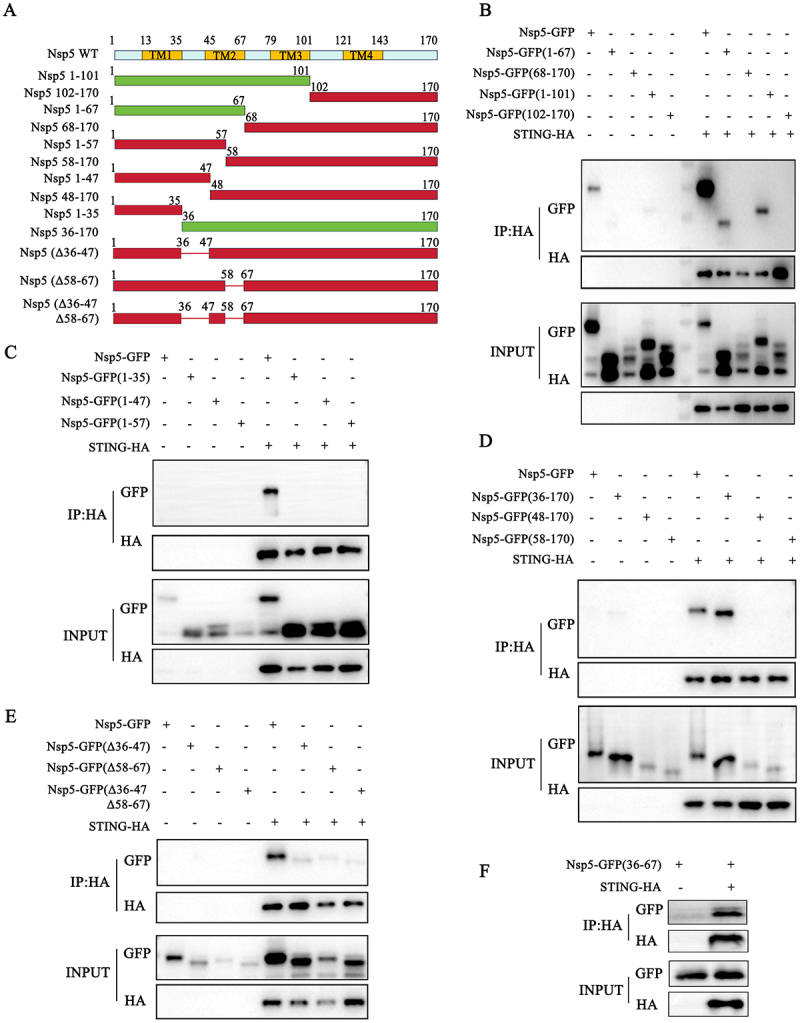
The 36–47 and 58–67 amino acids of Nsp5 are the key regions for interaction with STING. (A) A schematic of PRRSV Nsp5 molecular structure and its truncation and deletion mutants. (B-F) Nsp5-GFP or its truncation mutants/deletion mutants were co-transfected with STING-HA in HEK-293T cells for 24 h, and the cells were harvested and subjected to Co-IP and subsequent Western blot analysis using indicated antibodies.

To identify the region where Nsp5 interacts with STING, we prepared ten truncated Nsp5 mutants: 1–101, 1–67, 1–57, 1–47, 1–35, 102–170, 68–170, 58–170, 48–170 and 36–170 (Figure 7(A)). Co-IP assay results revealed that STING interacts with Nsp5 1–101 and 1–67, but not with Nsp5 68–170 and 102–170 (Figure 7(B)). Furthermore, STING did not interact with Nsp5 1–57, 1–47 and 1–35 (Figure 7(C)). These data indicated that 58–67 amino acids of Nsp5 are a necessary region for interaction with STING. On the other hand, STING interacted with Nsp5 36–170, but not with Nsp5 48–170 and 58–170 (Figure 7(D)). The results indicated that 36–47 amino acids of Nsp5 are also necessary for interaction with STING.

To ascertain whether the Nsp5 36–47 and 58–67 amino acid regions of Nsp5 are important for Nsp5 interaction with STING, two single and one double deletion mutants of GFP-tagged Nsp5 were prepared (Δ36–47, Δ58–67 and Δ36–47Δ58–67). Co-IP and confocal microscopy analysis showed that the regions of 36–47 and 58–67 amino acids are important for the binding and co-localization of Nsp5 to STING (Figures 7(E) and S2). In addition, we constructed the 36–67 amino acid region of Nsp5 covering these two regions, and the Co-IP and confocal microscopy results showed that STING interacts with the 36–67 region of Nsp5 (Figures 7(F) and S2), suggesting that the two regions are key regions for Nsp5 interaction with STING.

### The 36–47 and 58–67 amino acid regions of Nsp5 are crucial for interfering with STING signaling and PRRSV replication

**Figure 8.**
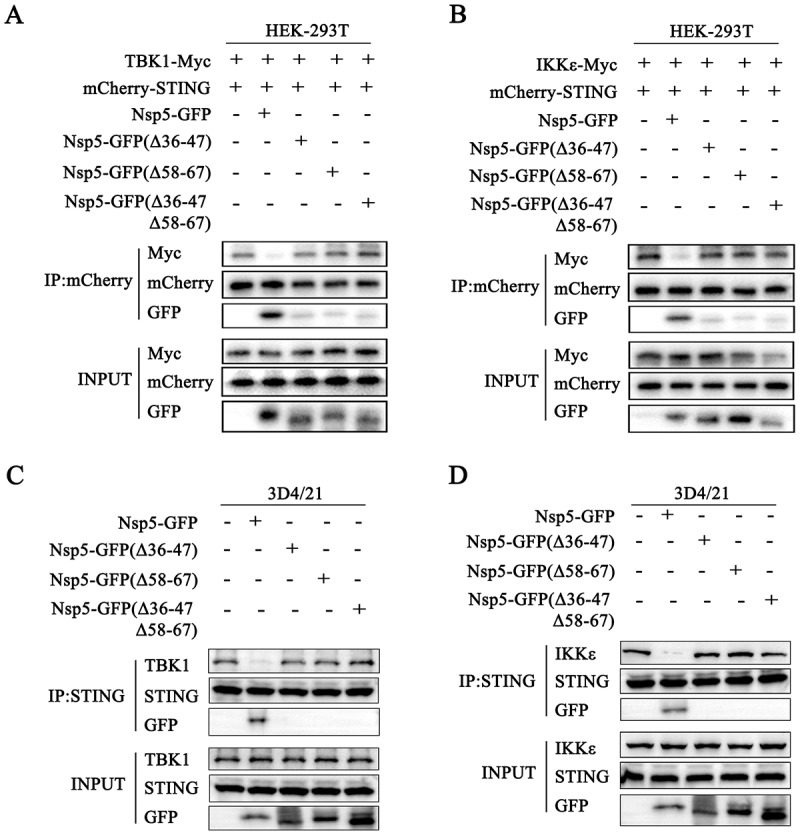
The regions of 36–47 and 58–67 amino acids of Nsp5 are crucial for interfering with STING signaling. (A) HEK-293T cells were co-transfected with TBK1-Myc together with mCherry-STING, Nsp5 or its deletion mutants for 24 h. (B) HEK-293T cells were co-transfected with IKKε-Myc together with mCherry-STING, Nsp5 or its deletion mutants for 24 h. Cell lysates were immunoprecipitated with anti-mCherry antibody and subjected to Co-IP and subsequent Western blot analysis. (C-D) 3D4/21 cells were transfected with Nsp5 or its deletion mutants for 24 h, and then stimulated with 2’3’-cGAMP for 12 h. Cell lysates were immunoprecipitated with anti-STING antibody and then analyzed by Western blotting for STING, TBK1, IKKε and GFP.

**Figure 9.**
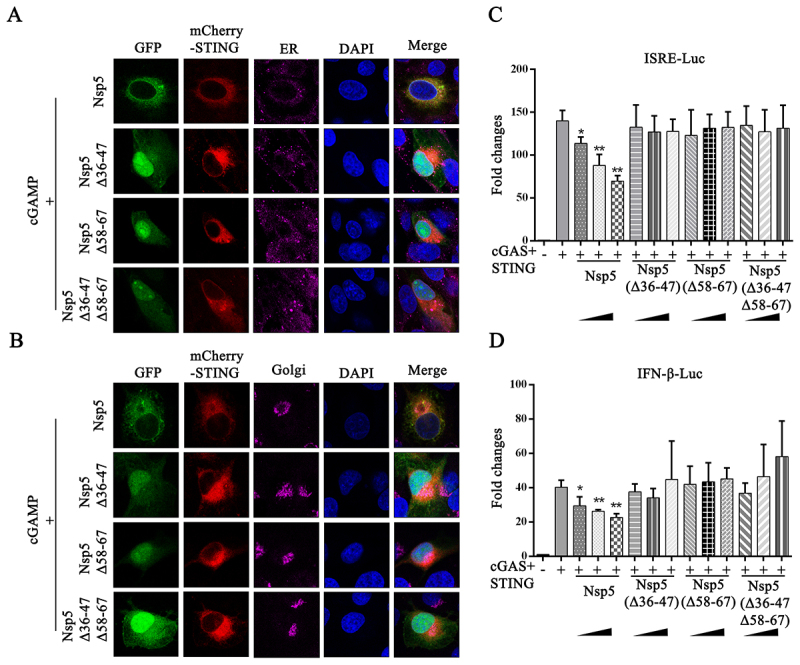
The regions of 36–47 and 58–67 amino acids of Nsp5 are crucial for blocking the STING trafficking and signaling. (A-B) 3D4/21 cells were co-transfected with STING and Nsp5 or Nsp5 deletion mutants and then treated with 2’3’-cGAMP. Cells were fixed, permeabilized and incubated with antibodies specific for calreticulin (A) and GORASP2 (B), followed by DAPI staining. Samples were examined under a confocal microscope. (C-D) HEK-293T cells were transfected with the ISRE-Luc (C), IFNβ-luc (D) reporter plasmid, together with Rluc reporter, cGAS, STING and Nsp5 or Nsp5 deletion mutant plasmids. Cells were collected 24 h after transfection, and ISRE/IFNβ promoter activity was evaluated via a luciferase reporter assay. * *p* < 0.05, ** *p* < 0.01.

We further investigated the impact of three deletions (Δ36–47, Δ58–67 and Δ36–47Δ58–67) in Nsp5 on the recruitment of TBK1 and IKKε by STING. The Co-IP results showed that, compared with full-length Nsp5, three deletion mutants lost the ability to disturb the interaction of exogenous STING and TBK1 (Figure 8(A)), and the interaction of exogenous STING and IKKε (Figure 8(B)). Consistently, three deletion mutants of Nsp5 also lost the ability to disturb the interactions of between endogenous STING and TBK1/IKKε (Figure 8(C,D)). Subsequently, we determined the effect of the three Nsp5 deletions on STING trafficking. Upon 2’3’-cGAMP stimulation, none of the Nsp5 deletion mutant blocked STING translocation from the ER to the Golgi apparatus (Figure 9(A,B)). Furthermore, compared with the full-length Nsp5, all three deletion mutants lost the ability to inhibit ISRE and IFNβ promoter activity (Figure 9(C,D)) as well as downstream ISG56, ISG60 and IFNβ gene productions (Figure S3) induced by the cGAS-STING.

To further clarify the importance of the 36–47 and 58–67 amino acids of Nsp5 in PRRSV infection, we also attempted to generate recombinant PRRSVs carrying the three deletion mutations by mutagenesis of the infectious cDNA clones (Figure S4A-B). Cytopathic effects were observed at 48 h post infection (hpi) in the recombinant PRRSV XJ17-5 (rXJ17–5) and recombinant PRRSV XJ17-5-EGFP (rXJ17-5-EGFP) infected Marc-145 cells, but not in cells mock-infected or infected with recombinant PRRSVs carrying any deletion mutations (Figure S4C). In addition, the GFP signal was visualized under a fluorescence microscope at 48 hpi in cells infected with rPRRSV XJ17-5-EGFP, but not in cells infected with rPRRSV XJ17-5 or recombinant PRRSVs carrying deletion mutations (Figure S4C). Together, these results demonstrated the critical roles of 36–47 and 58–67 amino acids of Nsp5 not only in blocking STING antiviral signaling, but also in PRRSV infection.

## Discussion

PRRSV is thought to be recognized by the innate immune RNA-sensing receptors TLR3/7/8/9 and RLRs [30]. In our previous study, we systematically dissected the anti-PRRSV activities of nine porcine innate immune signaling adaptors [28]. These results indicated that multiple porcine PRR signaling pathways might be involved in the sensing and fighting of PRRSV [28]. Furthermore, we and another group clearly demonstrated that the DNA-sensing cGAS-STING pathway plays an important role in sensing and counteracting PRRSV infection [21,31]. However, the interplay between PRRSV and DNA sensor-related host defense is intricate and the mechanism of viral evasion remains incompletely understood. In the present study, we identified a previously unrecognized mechanism by which PRRSV inhibits porcine STING antiviral signaling by blocking its trafficking. This viral immune evasion is dependent on the viral protein Nsp5, which interacts with STING in the ER to impair STING transport to the Golgi apparatus, and in turn affects downstream antiviral immunity. This study provides a better understanding of the immune escape mechanism and pathogenesis of PRRSV.

During PRRSV infection, there is a complex relationship between the host immune defense and viral infection [32–34]. Our data strongly support the conclusion that Nsp5-mediated retention of STING in the ER blocks its ability to engage canonical downstream signaling partners TBK1/IKKε/IRF3. However, the fine mechanistic basis of this interference remains somewhat underexplored. For example, it is unclear whether Nsp5 physically anchors STING in the ER membrane, disrupts its palmitoylation, or modulates host trafficking machinery. Indeed, STING activation depends on its palmitoylation, which facilitates its trafficking from the ER to the Golgi apparatus [35], warranting the further investigation. Interestingly, a recent study showed that the Nsp2 of PRRSV impedes STING translocation from the ER to the Golgi apparatus by deubiquitinating STIM1 via Nsp2 deubiquitinating (DUB) activity [36], thereby facilitating immune escape. We did not observe an interaction between Nsp2 and STING in our initial screening experiments, which may be due to the various experimental conditions. Nevertheless, it is possible that several PRRSV non-structural proteins (Nsps) are involved in evasion of STING trafficking and signaling. Further exploration is needed to provide deeper insights into the evasion of STING traffic and signaling by PRRSV.

There are relatively few studies on Nsp5, which is a conserved non-structural protein of PRRSV composed of approximately 170 amino acid residues [37]. Nsp5 is a hydrophobic transmembrane protein that forms a membranous structure in the cytoplasm, which could be the site for viral replication [1]. Nsp5 participates in the formation and maintenance of double-membrane vesicles (DMV) during PRRSV infection, thereby promoting the synthesis and replication of viral RNA [38]. Also, Nsp5 promotes the formation and accumulation of autophagosomes [39,40]. In terms of immune regulation, Nsp5 antagonizes JAK/STAT3 signaling by inducing the degradation of STAT3 [41], and it also inhibits the RLR signaling pathway by degrading multiple proteins of this pathway [42]. Therefore, Nsp5 not only antagonizes the RNA sensor mediated innate immune response, but also the DNA sensor mediated innate immune response.

In this study, we identified two key regions of Nsp5 that interact with STING and attempted to generate recombinant viruses carrying Nsp5 deletion mutations using mutagenic infectious cDNA clones. However, it failed to rescue the recombinant viruses, likely because of the large number of deleted amino acids, highlighting the critical role of Nsp5 in PRRSV replication. Currently, we are attempting to identify the key amino acids that interact with STING and rescue recombinant viruses carrying point mutations in Nsp5, which will be useful for investigating the impact of Nsp5 on innate immunity in *vitro* and *in vivo*.

**Figure 10.**
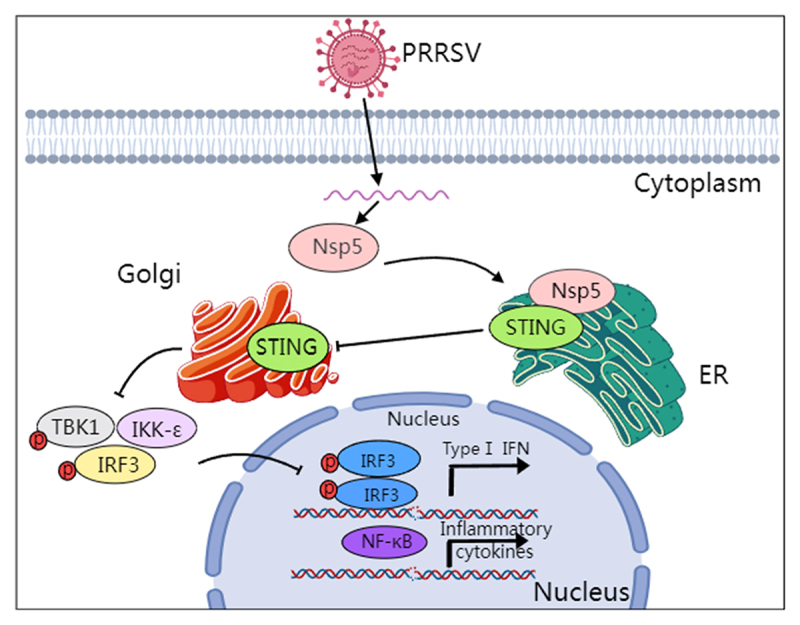
Schematic model of the mechanism by which PRRSV Nsp5 inhibits the cGAS-STING signaling pathway, created in MedPeer. The interaction between PRRSV Nsp5 and STING hinders the transport of STING from ER to Golgi apparatus, and thus disrupts the assembly of the STING/TBK1/IKKε/IRF3 signaling complex and downstream antiviral IFN response.

In conclusion, this study revealed that PRRSV Nsp5 can inhibit the DNA innate immune cGAS-STING antiviral signaling pathway, by blocking porcine STING trafficking from the ER to the Golgi apparatus and interfering with recruitment of TBK1/IKKε/IRF3 (Figure 10). Importantly, the 36–47 and 58–67 amino acids of Nsp5 are the key regions for interacting with porcine STING, immune escape and PRRSV replication. These findings provide new insights into the pathogenesis of PRRSV and development of novel antiviral strategies against PRRSV.

## Data Availability

The authors confirm that the data supporting the findings of this study are available in the article and its supplementary materials. Supplementary Tables , raw WB and IFA data, and numeral raw data are all available online: https://doi.org/10.6084/m9.figshare.28716005
